# Spatial Price Transmission and Dynamic Volatility Spillovers in the Global Grain Markets: A TVP-VAR-Connectedness Approach

**DOI:** 10.3390/foods13203317

**Published:** 2024-10-18

**Authors:** Huidan Xue, Yuxuan Du, Yirui Gao, Wen-Hao Su

**Affiliations:** 1 College of Economics and Management, Beijing University of Technology, Beijing 100124, China; huidan.xue@bjut.edu.cn; 2 Beijing-Dublin International College at BJUT, Beijing University of Technology, Beijing 100124, China; duyuxuan@emails.bjut.edu.cn (Y.D.);; 3 College of Engineering, China Agricultural University, Beijing 100083, China

**Keywords:** global grain markets, grain prices, price transmission, volatility spillover, TVP-VAR-Connectedness model

## Abstract

The global food market’s escalating volatility has led to a complex network of uncertainty and risk transmission across different grain markets. This study utilizes the Time-Varying Parameter Vector Autoregression (TVP-VAR)-Connectedness approach to analyze the price transmission and volatility dynamics of key grains, including wheat, maize, rice, barley, peanut, soybean, and soybean meal, and their dynamic spillover directions, intensity, and network. By integrating the TVP-VAR-Connectedness model, this research captures the time-varying variability and interconnected nature of global grain price movements. The main findings reveal significant spillover effects, particularly in corn prices, with prices of soybean dominating other grains while prices of peanut and corn experience higher external spillover effects from other grains. The conclusions drawn underscore the imperative for policymakers to consider a holistic perspective of all types of grains when addressing global food security, with this study providing valuable insights for risk management in the grain sector at both global level and country level.

## 1. Introduction

The volatility inherent in international grain prices garners significant attention from academics, policymakers, investors, farmers, and consumers alike. International grain prices wield considerable influence over grain security, bearing direct relevance to the essential welfare of local populations [1]. These price volatilities not only directly impact prices alone or along the supply chain but also transmit across different types of food and grains, engendering implications for overall price stability. Since 1998, international grain prices have experienced prolonged and significant fluctuations, particularly during the global food crisis of early 2010, the outbreak of the COVID-19 pandemic during the year of 2020 to 2022, and the Russia–Ukraine conflict since 2022. The contagious volatility observed in international grain prices has introduced profound uncertainty into the global grain market, posing threats not only to global grain security but also to economic and social stability of many countries, especially those with import reliance [2,3]. Consequently, nations worldwide accord significant importance to monitoring the evolving trend and transmission pattern of international grain prices, deeming the stabilization of grain prices as a pivotal objective of economic and social policies. In recent years, the influence of fluctuations in international grain prices on local grain markets have ignited profound concerns, particularly regarding the global food price hikes in many developing nations. Countries in development that rely on grain imports are often subject to sharp price increases, which can precipitate local food inflation and pose risks to food security. Anon (2020) [4] finds that the rapid rise in international grain prices due to the COVID-19 pandemic has led to a significant increase in local grain prices in many underdeveloped countries, endangering the food security of over 100 million people. Therefore, comprehending the transmission dynamics and volatility spillovers of different grain prices in global markets assumes heightened significance in formulating effective policies aimed at addressing global food security, especially for those countries that are more vulnerable to global price surges.

In fact, the phenomenon of price transmission and the volatility spillovers within agricultural commodities exhibit an interdependence. This interdependence is characterized by the propensity for price fluctuations in a specific agricultural commodity to transmit rapidly across contiguous markets, thereby inciting a cascading effect that influences the stability and predictability of the global food supply chain. Therefore, the price surges of grains have never been only for a single type of grain. For instance, an escalation in the pricing of maize has the potential to exert a significant impact on the energy sector by augmenting the production costs of biofuels, and concurrently, it may precipitate a ripple effect on the pricing mechanisms of other crops, such as wheat, through the economic principle of substitution effects [5,6]. As maize is increasingly redirected to biofuel production instead of being used for food or animal feed, food grains like wheat and soybeans are being substituted in animal feed and human consumption. The demand for corn-based ethanol impacts soybeans, as both crops compete for land and are often grown in rotation [2].

In recent years, the risk of spillover effects has not subsided, and agricultural commodity markets remain notably vulnerable to external shocks. International grain prices are significantly influenced by a range of factors, including global demand dynamics, weather fluctuations in key exporting nations, and speculative activities within commodity markets. The emergence of the current global food crisis does not come as a surprise, given historical precedents. Past global food crises may have been attributed to various factors, including the following: (1) Surging oil prices from 2007 to 2008 led to a price surge in grain prices, especially maize. (2) Escalating food demand in emerging economies [7,8] has precipitated an escalation in both the demand for and the pricing of food commodities. (3) There have been occurrences of extreme weather events such as droughts, floods, and storms in key grain-producing nations such as China, Australia, Argentina, Russia and Ukraine during the years 2010–2011 [9], and this inclination to store foodstuffs escalates, consequently leading to an increased demand and subsequent price surge in cereals and grains. (4) Disruptions in global food supply chains caused by the COVID-19 pandemic [10,11,12] and (5) the rising food prices driven by the Russia–Ukraine conflict [13,14,15] have led to significant price surges and fluctuations due to their impacts on production, supply, and consumption. The above events led to price surges or fluctuations due to its effects on production, supply, and consumption. Owing to the aforementioned series of events and economic effects, there is a persistent fluctuation in the uncertainty surrounding the demand for and pricing of grains, which connotes a concomitant oscillation in risk. Additionally, the adverse effects of the Russia–Ukraine conflict on upstream energy and fertilizer markets are likely to reverberate through the food market, potentially exacerbating food insecurity in specific countries and regions [15]. Increased transportation costs may further contribute to food insecurity in nations heavily dependent on imports from conflict-affected areas [16]. The decline in food exports from Russia and Ukraine, combined with soaring energy prices, could result in a 60–100% increase in food costs by 2023 compared to 2021 [17]. Since the start of the Russia–Ukraine conflict, many countries have imposed export restrictions, while excessive futures speculation has led to sharp increases and dramatic fluctuations in international food prices [18]. The episodes above highlight the necessity of restructuring agri-food systems to enhance resilience and effectively address such crises.

This paper aims to advance the field of spatial price transmission research by examining the dynamics of spatial price transmission and the volatility spillovers observed in global grain markets. It focuses on the interactions and directional characteristics of price transmission and volatility spillovers across various grain types. The TVP-VAR-Connectedness approach [19], with different dynamic connectedness indices of the grain systems illustrated, is employed in this study to figure out the complex linkages and spillover effects of prices of different types of grains in international grain markets. This research aims to yield invaluable insights for stakeholders from market participants to policymakers, providing guidance in navigating the complexities inherent to global grain markets and effectively mitigating associated risks. Furthermore, it offers insights for producers, consumers, and investors regarding the utilization of different grain prices as signals to mitigate food price risks. Our findings have significant implications for policymakers, aiding in the development of food security trade policies designed to mitigate agricultural price risks within the grain markets of developing economies.

The subsequent sections of this paper are organized as follows: Section 2 offers a thorough review of existing studies on grain price transmission. Section 3 outlines the research methodology and data used. Section 4 presents the empirical findings and analysis, while Section 5 discusses the conclusions drawn from these findings and offers implications for policy and future research.

## 2. Literature Review

Previous research has predominantly revolved around the spatial price transmission effects within the same type of grain in different countries, often employing VAR (Vector Autoregression) or VEC (Vector Error Correction) types of models. Furthermore, the utilization of various GARCH (Generalized Autoregressive Conditional Heteroskedasticity) models has facilitated the research on examining the volatility spillover effects between different types of grains, thereby illuminating the intricate dynamics of price movements and volatility spillovers within and across markets. As research into grain price transmission has advanced, researchers have increasingly employed sophisticated Generalized Autoregressive Conditional Heteroskedasticity (GARCH) models to investigate the mechanisms underlying price fluctuations across different grain varieties, primarily focusing on the variance in price series. The TVP-VAR model can be utilized to analyze the time-varying net return linkages between variables, revealing the volatility linkages evolve over time, particularly intensifying during crises. While GARCH, BEKK-GARCH, and DCC-GARCH models are useful for analyzing volatility spillovers in energy and food prices, they are often critiqued for their complexity, limited ability to handle non-linearity and asymmetry, over-reliance on historical data, and risk of overfitting.

Using both a BEKK and a DCC model, Hernandez et al. (2014) [20] highlighted significant volatility spillovers within corn, wheat, and soybean futures markets across the United States, Europe, and Asia. Beckmann and Czudaj (2014) [21] examined short-term volatility spillover effects between wheat, corn, and cotton using a bivariate GARCH-in-mean VAR model. Gardebroek et al. (2016) [22] applied various MGARCH models to analyze grain price data at different frequencies, suggesting that at the price level, the price transmission between corn, wheat, and soybean is not significant. Nevertheless, in terms of volatility spillover effects, there are significant impacts between corn, wheat, and soybean prices at weekly and monthly frequencies. In recent years, similar research has also been conducted for domestical price transmission and volatility effects. Rezitis et al. (2024) [23] integrated the DCC-MVGARCH model, the Markov-switching regression approach, and the BEKK-MVGARCH model into a single framework to identify subperiods of low and high conditional cross-correlations between energy and agricultural futures returns. Moreover, Yang and Karali (2022) [24] found that the volatility response of Chinese soybean product markets to innovations based on the U.S. soybean weakened after 2009, using a multivariate GARCH model with BEKK specification. Zhao and Goodwin (2011) [25] identified significant volatility spillovers between corn and soybean futures prices using a BEKK model. Yosthongngam et al. (2022) [26] employed both BEKK and DCC models to uncover volatility spillovers within corn, wheat, and soybean futures exchanges across various regions, alongside a marked increase in interdependence over time. Significant bidirectional volatility spillovers were observed between corn and ethanol across all countries, with the co-volatility between their returns showing instability and fluctuations over time.

The existing literature presents rich insights on the spatial transmission of grain prices within the context of developing countries. Conforti (2004) [27] examined price transmission across 16 countries, with a particular focus on Sub-Saharan Africa, and found variations in the degree of transmission when compared to Asian and Latin American nations. The long-term relationship between world grain markets and local food prices was further supported by Baquedano and Liefert (2014) [28], who identified such relationships in the majority of 61 local prices tested. More recent studies have expanded the analytical scope; for instance, Garcia-German et al. (2016) [29] employed error correction models to assess price transmission between global agricultural markets and consumer food price indices within European Union member states.

International grain markets have witnessed pronounced volatility, with the pricing dynamics of grains being influenced by various macroeconomic determinants, including but not limited to supply and demand dynamics, extreme turbulent events, production costs, natural calamities, and climate-related risks. Živkov, Njegić, and Pećanac (2019) [30] employed wavelet methodologies to investigate the multiscale dynamic interlinkages among wheat, corn, soybean, oats, and rice. Frimpong et al. (2021) [31] delved into the temporal-frequency ramifications of global economic policy uncertainty on the interconnections among oats, rice, corn, wheat, and soybean, leveraging wavelet techniques. Their analyses found significant temporal and frequency-dependent disparities in spillover effects within agricultural markets. Guo et al. (2023) [32] innovatively constructed three distinct climate risk perception indices to assess their divergent impacts on bulk agricultural prices. Their findings underscored that agricultural product prices exhibit more pronounced responses to climate risk perceptions in the short term as opposed to the long term. The COVID-19 pandemic resulted in market uncertainty, heightened distribution costs, sparked panic-driven demand, and significantly increased the short-term price volatility of agricultural commodities [33,34]. The Russian–Ukrainian conflict threatened the world’s food security in various ways, including creating challenges for production, costs, and international trade. Zhou et al. (2024) [13] introduced an innovative analytical framework for assessing tail dependence. This approach facilitated an exploration of tail dependence structures and extreme risk spillovers between agricultural futures and spot markets before and after the outbreak. Their findings highlighted that tail dependence structures in the futures–spot markets for soybean, maize, wheat, and rice have all been affected by the Russia–Ukraine conflict, resulting in varying levels of amplified risk across these agricultural markets.

Overall, the existing literature provides a comprehensive overview of price transmission mechanisms among various market pairs, encompassing intra-national dynamics, interactions between international and local markets, and interconnections among diverse international commodity prices. Furthermore, an expanding body of literature delves into the transmission of price volatility and its determinants. However, these methods could not provide the visual and dynamic spillover effects of grain price systems in terms of direction, intensity, network relationship, and connectedness in the global grain systems. To the best of our knowledge, scant attention has been devoted to investigating the direction, intensity, and time-varying effects of spillover effects and the network dynamics in the context of international grain price fluctuations. It is this gap in the literature that we endeavor to bridge through this study.

## 3. Methods and Data

### 3.1. Method

The TVP-VAR-Connectedness approach is employed in this paper.

Following the study of Antonakakis et al. (2020) [35], the TVP-VAR model with lag length *p*, which is determined by the Bayesian information criterion (BIC), can be defined as follows:

(1) $$y_{t}=A_{t}z_{t-1}+\varepsilon_{t}\cdots \cdots \varepsilon_{t}|\Omega_{t-1}∼\;N(0,\Sigma_{t})$$

(2) $$vec(A_{t})=vec(A_{t-1})+\xi_{t}\cdots \cdots \xi_{t}|\Omega_{t-1}\;\sim N(0,\Xi_{t})$$

(3) $$z_{t-1}=\left(\begin{array}{c} y_{t-1}\\ \cdots \\ y_{t-p} \end{array}\right),=\left(\begin{array}{c} A_{1t}\\ \cdots \\ A_{pt} \end{array}\right)$$

Here, $\Sigma_{t}\;and\;\Xi_{t}$ are the time-varying variance–covariance matrix with *m* × *m* and *m*^2^*p* × *m*^2^*p* dimensions. $\Omega_{t-1}$ encapsulates all the information available till t − 1. *vec*($A_{t}$) is a vector of $A_{t}$ with *m*^2^*p* × 1 dimensions. Similarly, $Z_{t-1}$ and error term $\;\varepsilon_{t}$ denotes the vector with *k* × 1 dimensions.

Employing the time-varying coefficient and variance–covariance matrices, we calculate the generalized connectedness proposed by Diebold and Yılmaz (2014) [36], which depends on the generalized impulse response functions (GIRFs) and generalized forecast error variance decompositions (GFEVDs). For the above purpose, we convert the TVP-VAR into vector moving average representation, as given below:

(4) $$y_{t}=J’\;(M_{t}(z_{t-2}+\eta_{t-1})+\eta_{t}=J’\;(M_{t}(M_{t}(z_{t-3}+\eta_{t-2})+\eta_{t-1}+\eta_{t})=J’\;(M_{t}^{k-1}z_{t-k-1}+\sum_{j=0}^{k}M_{t}^{j}\eta_{t-j})$$

(5) $$M_{t}=\left(\begin{array}{cc} A_{t} & \\ I_{m(p-1)} & 0_{m(p-1)\times m} \end{array}\right)\;\eta_{t}=\left(\begin{array}{c} \varepsilon_{t}\\ \cdots \\ 0 \end{array}\right)=J\varepsilon_{t}\;j=\left(\begin{array}{c} I\\ \cdots \\ 0 \end{array}\right)$$

where $M_{t}$ is a *mp × mp* dimensional matrix, $\eta_{t}$ is a *mp* × 1 dimensional vector, and *J* is an *mp × m* dimensional matrix. As k approaches ∞, this yields

(6) $$y_{t}=\;\underset{k\to \infty }{\lim}J’(M_{t}^{k-1}z_{t-k-1}+\sum_{j=0}^{k}M_{t}^{j}\eta_{t-j})\;=\sum_{j=0}^{\infty }{J’M}_{t}^{j}\eta_{t-j}$$

where it can be expressed as follows:

(7) $$y_{t}\;=\sum_{j=0}^{\infty }{J’M}_{t}^{j}J\varepsilon_{t-j}\;\;\;B_{jt}={J’M}_{t}^{j}J,\;\;\;j=0,\;1,\;...$$

(8) $$y_{t}=\sum_{j=0}^{\infty }\;B_{jt}\varepsilon_{t-j}$$

where $B_{jt}$ is a matrix of *m × m* dimensions.

$\psi_{ij,t}(H)$ represents the response of all variables *j* to a shock in variable *I*. Since the model is non-structural, we therefore use *H*-step-ahead forecast model, which is as follows:

(9) $${GIRF}_{t}(H,\delta_{j,t},\Omega_{t-1})\;=E(y_{t+H}|e_{j}=\delta_{j,t},\Omega_{t-1})-E\left(y_{t+J}|\Omega_{t-1}\right)$$

(10) $$\psi_{j,t}(H)=\frac{B_{H,t}\sum_{t}e_{j}}{\sqrt{\sum_{jj,t}\;}}\;\frac{\delta_{j,t}}{\sqrt{\sum_{jj,t}\;}}\;\;\;\;\delta_{j,t}=\sqrt{\sum_{jj,t}\;}\;$$

(11) $$\psi_{j,t}(H)=\sum_{jj,t}^{-\frac{1}{2}}B_{H,t}\sum_{t}e_{j}\;\;\;$$

Here, $e_{j}$ denotes a *m* × 1 selection matrix with 1 on the jth position, and zero otherwise. Next, we compute the GFEVD ${\tilde{\;(\varphi }}_{ij,t}$(*H*)) according to Equation (12), which represents the pairwise directional connectedness from *j* to *i* and illustrates the influence variable *j* has on variable *i* in terms of its forecast error variance share. These variance shares are then normalized, so that each row sums up to one, meaning that all variables together explain 100% of variable *i*’s forecast error variance.

(12) $${\tilde{\varphi }}_{ij,t}(H)=\frac{\sum_{t=1}^{H-1}\psi_{ij,t}^{2}}{\sum_{j=1}^{m}\sum_{t=1}^{H-1}\psi_{ij,t}^{2}}$$

We construct the total connectedness index (TCI) by Equation (13).

(13) $$C_{t}\left(H\right)=\frac{\sum_{i,j=1,i\neq j}^{m}{\tilde{\varphi }}_{ij,t}\left(H\right)}{\sum_{i,j=1}^{m}{\tilde{\varphi }}_{ij,t}\left(H\right)}\times 100=\frac{\sum_{i,j=1,i\neq j}^{m}{\tilde{\varphi }}_{ij,t}(H)}{m}\times 100$$

This connectedness approach shows how a shock in one variable spills over to other variables. First, we look at the case where variable *i* transmits its shock to all other variables *j*, called total directional connectedness to others (TO) and defined as

(14) $$C_{j\leftarrow i,t}(H)=\frac{\sum_{j=1,j\neq i}^{m}{\tilde{\varphi }}_{ij,t}(H)}{\sum_{i=1}^{m}{\tilde{\varphi }}_{ij,t}(H)}\times 100$$

Second, we calculate the directional connectedness variable *i* receives it from variables *j*, called total directional connectedness from others (FROM) and defined as

(15) $$C_{i\leftarrow j,t}\left(H\right)=\frac{\sum_{j=1,j\neq i}^{m}{\tilde{\varphi }}_{ij,t}(H)}{\sum_{j=1}^{m}{\tilde{\varphi }}_{ij,t}(H)}\times 100$$

Furthermore, we subtract total directional connectedness to others (TO) from total directional connectedness from others (FROM) to obtain the net total directional connectedness (NET), which can be interpreted as the influence variable *i* has on the analyzed network.

(16) $$C_{i,t}(H)=C_{j\leftarrow i,t}(H)-C_{i\leftarrow j,t}(H)$$

If the net total directional connectedness (NET)of variable *i* is positive, it means that variable *i* influences the network more than being influenced by that. By contrast, if the net total directional connectedness is negative, it means that variable *i* is driven by the network.

Finally, we further decompose the net total directional connectedness (NET). By calculating the net pairwise directional connectedness (PAIR) to examine bidirectional relationships,

(17) $$NPDC_{ij}\left(H\right)={(\tilde{\varphi }}_{jit}(H)-{\tilde{\varphi }}_{ijt}(H))\times 100.$$

If $NPDC_{ij}(H)$ > 0 ($NPDC_{ij}(H)$ < 0), it means that variable *i* dominates (is dominated by) variable *j*.

### 3.2. Data

This study focuses on the markets of different agricultural commodities, namely wheat, maize, rice, barley, peanut, soybean, and soybean meal. The empirical analysis is conducted utilizing a time series dataset comprising monthly price observations spanning from 31 January 2001 to 31 December 2023. The dataset for agricultural prices is sourced from the International Grains Council (IGC). These prices offer a robust representation of the fluctuating dynamics in international agricultural spot prices. The analytical framework of this research is anchored in the Time-Varying Parameter Vector Autoregressive (TVP-VAR) model, which is estimated employing the Bayesian Information Criterion (BIC) to ensure optimal model specification. The forecasting horizon for the TVP-VAR model is set at 100 periods, aligning with the temporal resolution of the dataset and the research objectives. Concurrently, the dynamic spillover indices, which are pivotal for assessing the interdependence and transmission of shocks across the commodity markets, are estimated employing a rolling window approach of 260 periods, coupled with a forecasting horizon of 100 period. This methodological approach allows for an examination of the time-varying evolution and cross-market spillovers within the grain systems.

## 4. Results and Analysis

### 4.1. Price Dynamics

**Figure 1:**
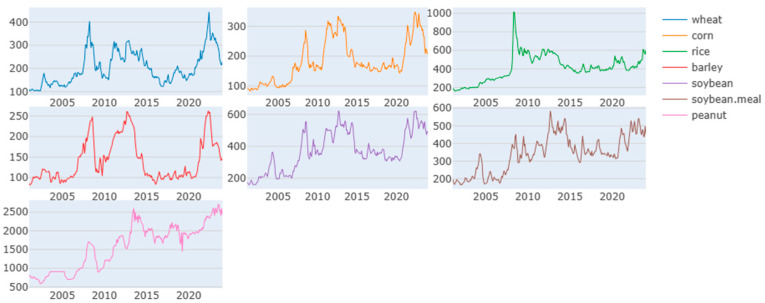
Prices of selected grains from January 1980 to December 2023.

The graph depicted in Figure 1 illustrates the monthly price dynamics characterizing the evolution of key agricultural commodities: wheat, corn, rice, barley, soybean, soybean meal, and peanut. Our analysis is confined to the period spanning from January 2004 to December 2023. Noteworthy observations emerge from Figure 1: the prices of different grains exhibit a similar pattern over the years in the analysis period.

These grains underwent a significant price surge in the global financial crisis of 2007–2008 and in the food crisis of 2009, likely instigated by heightened biofuel demand, escalating oil prices, and the depreciation of the U.S. Dollar [37,38]. The increasing prominence of biofuels emerged as a pivotal factor contributing to the escalation in food commodity prices, alongside the amplifying influence of rising energy costs and the devaluation of the U.S. dollar relative to major global currencies [37]. Moreover, these grain price series experienced notable price volatility during the European debt crisis from 2011 to 2012. Currency depreciation and tightened liquidity may have precipitated adjustments in consumer consumption patterns [39,40,41]. Significantly, amid the COVID-19 pandemic spanning from 2019 to 2022, these commodities witnessed multiple sharp price peaks. Notably, before 2020, wheat, corn, and barley exhibited similar variations, likely due to the cumulative effects of the Russia–Ukraine conflict [42]. As the largest and fifth-largest wheat exporters globally, Russia and Ukraine accounted for 16.07% and 9.18% of total global wheat exports, respectively. Ukraine ranks third and Russia fourth in barley export volumes, representing 16.78% and 13.02% of total global barley exports, respectively. Both countries also play significant roles as major corn exporters, with Ukraine and Russia contributing 13.94% and 2.07% of total global maize exports, respectively [13]. Given the crucial roles of Russia and Ukraine in the global food supply, the outbreak of the Russia–Ukraine conflict has undeniably had a significant impact and presents a considerable risk to the global food system [13,43,44]. All three events created economic instability that disrupted global supply and demand for grains. The financial crisis and the European debt crisis largely caused demand-side shocks, leading to a collapse in commodity prices due to reduced economic activity. In contrast, the Russia–Ukraine conflict has caused supply-side shocks, particularly in energy and agriculture, leading to price surges and shortages. In conclusion, these crises are interconnected through their cumulative effects on global demand, supply chains, and market vulnerabilities, leading to significant volatility and systemic risks in the global grain markets.

It is necessary to conduct an Augmented Dickey–Fuller (ADF) unit root test on the price data of wheat, corn, rice, barley, soybean, soybean meal, and peanut prices to check the stationarity of the analyzed time series data. Table 1 reveals the statistics of the ADF test results of the level and first difference for all seven series. The *p*-values of level prices are not significant, while the *p*-values of the first difference prices are zero, which means that all the prices have one unit root. Consequently, it can be affirmed that the first difference of the examined time series exhibits stationarity, wherein their statistical attributes such as mean and variance remain constant over time. Therefore, it is suitable to conduct a TVP-VAR analysis using first difference prices.

The descriptive statistics are in Table 2. Rice and soybean have the highest and lowest volatility values, respectively.

The results show that the price series are significantly skewed, with all exhibiting right skewness and excess kurtosis. The JB estimates indicate that all series are sharply peaked, confirming their non-normal distribution. These findings support the use of the interdependencies model for all series through the application of the TVP-VAR technique [45,46].

**Figure 2:**
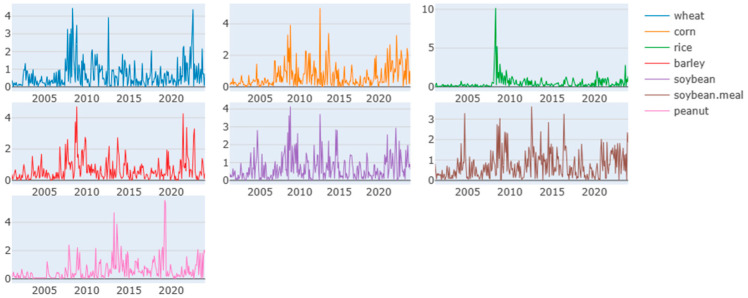
Standardized first differences series of key agricultural commodity prices from July 2001 to December 2023.

Figure 2 illustrates the evolution of standardized first differences for seven types of grains, showing a similar price change pattern throughout most of the analysis period. Notably, there was a significant increase during and after the 2007–08 food crisis, followed by a decline. Wheat, corn, and soybean displayed high price change during the European debt crisis (2011–2012) and the Russian–Ukrainian war post–2021. Additionally, peanut price changes peaked during the high biofuel demand period from 2013 to 2014 [47] and during the COVID-19 pandemic from 2019 to 2021.

### 4.2. Volatility Analysis

Table 3 displays the paired time-invariant price change connectedness between seven price series of grains. The grains included in this study are wheat, corn, rice, barley, soybean, soybean meal, and peanut. The analysis reveals a significant level of connectedness among all the prices studied, as indicated by the considerable value of 41.42% for the total connectedness index (TCI) of the network. The TCI is derived by dividing the sum of directional spillovers from and to the network by the directional spillovers and own effect, namely, 289.96 divided by 700. Specifically, the TCI stands at 41.42%, signifying a notable degree of interconnectivity among grains. Furthermore, the figures on the diagonal of the table represent price spillovers within each market, while the off-diagonal figures denote the directional price connectedness between pairs of grain prices in this investigation.

Each column illustrates the contribution of the relevant grain’s shock (to) the other grains of the grain systems. Each row provides the individual contributions of the shock (from) each grain to the relevant grain. In the last column of Table 3, the term “FROM” signifies the price connectedness from the specific grain of the system to all other prices. These spillovers are calculated by aggregating the off-diagonal spillover figures in each row. The findings reveal that soybean (58.59%), soybean meal (55.13%), and corn (50.78%) are the main receivers of shock. Peanut (12.81%) is identified as the grain with the least amount of price spillover received from the network, indicating the bottom-most shock receiver of the network. The connectedness value from each grain to the system of all other grain markets is depicted in the third row from the bottom, labeled “TO”. The findings disclose that soybean (75.87%), soybean meal (58.03%), and corn (47.96%) exhibit the most significant transmission of price spillovers to the overall system. Peanut (6.38%) is the market with the lowest transmission of price spillovers, suggesting a minor degree of price interconnectedness with the system. Finally, “NET” in the last row represents the net directional connectedness for the seven grain prices in this study. Negative (positive) numbers indicate that the grain is a net recipient (exporter) of connectedness from/to the network. In terms of net spillovers, wheat (−5.15%), corn (−2.82%), rice (−4.16%), barley (−1.62%), and peanut (−6.43%) are the net recipients of price spillovers, whereas soybean (17.29%) and soybean meal (2.89%) act as the net transmitters of price spillovers.

**Figure 3:**
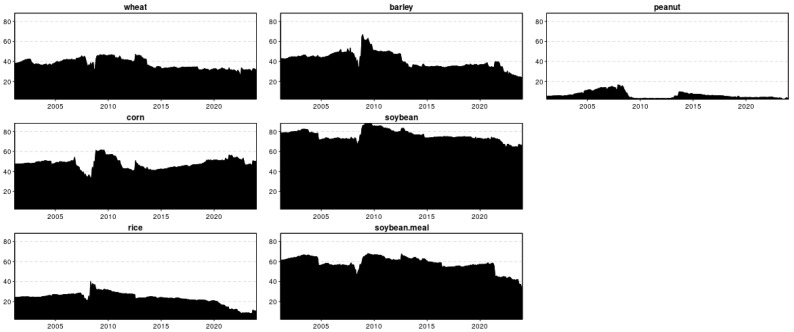
Time-varying total directional connectedness to others (TO).

**Figure 4:**
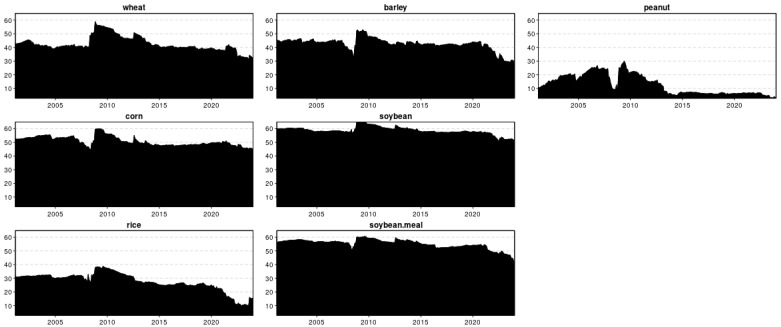
Time-varying total directional connectedness from others (FROM).

Figure 3 and Figure 4 present the amount of spillover that is transmitted to and received from all the other grains, respectively. In Figure 3, the time series plot of the spillover intensity of the seven grains shows high volatility and salient peaks, notably possessing peaks and changes that are synchronous during the turmoil periods of the 2008–2009 global financial crisis [37,48,49,50], and the food crisis of 2011–2012 [39,40,41], indicating that grain prices are more sensitive to unexpected news or turbulent events. Barley used to be a much less influential grain than wheat until the outbreak of the COVID-19 pandemic and Russia–Ukraine war. It can be observed that the most dynamic increase in the spillover index for barley was during the COVID-19 era from October 2020 to March 2023. In the time of 2022–2023, especially with the escalating Russia–Ukraine war, it transmits to and receives from others more spillovers than soybeans, which seemed to be a much more influential agricultural commodity so far [51]. An intercomparison of the spillover intensity of the previous grains showed that soybean prices dominated other grain prices, with a characteristic of the mean spillover intensity reaching 75.87%. At the same time, peanuts have the lowest, 6.38%.

Moreover, the spillover indices TO for wheat show a lower fluctuation frequency, indicating that they are more susceptible to ongoing market trends. In Figure 4, the following graph presents the FROM indices for the seven-time series. Analogous to the previously analyzed spillover index too, the frequency of changes in peanut is relatively higher than the other six indices, and its values are relatively lower, highlighting that market sentiment is less influenced by parts of the system and more by sudden events. The FROM indices of soybeans are generally above 50%, which can be explained by a higher total directional connectedness from others (FROM) index indicating that these indices are highly susceptible to spillover effects from the Chinese stock market system.

**Figure 5:**
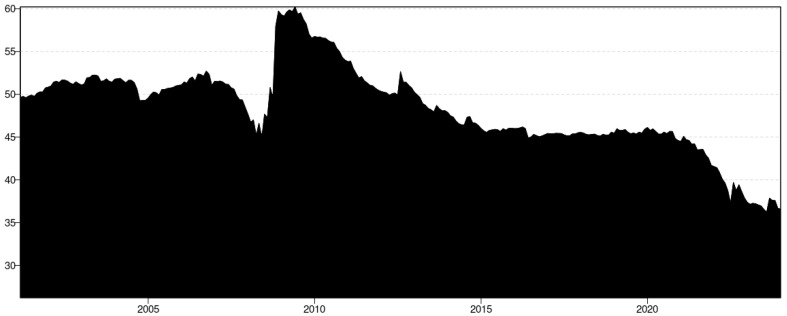
Dynamic total connectedness (TCI).

The total connectedness index (TCI) shown in Figure 5 quantifies the magnitude of price volatility spillovers within the examined network. The black-shaded area represents the TCI trend, accounting for both positive and negative returns. A key observation is the significant evolution of total connectedness in this network, with notable magnitudes reflected in the black-shaded region. Throughout the sample period, the TCI averaged 48.33%, demonstrating considerable volatility and fluctuating between approximately 35% and 60%. This TCI figure indicates a strong and time-varying interconnectedness among different grains, a dynamic often obscured by the static nature of the TCI.

Notably, during periods of economic upheaval, such as the global food crisis of 2007–2008, the TCI consistently maintained levels exceeding 44% when the maximum TCI value surpassed 60%, suggesting that volatility in food prices may engender heightened connections amidst major economic shocks. The second noteworthy period is the food crisis from 2011 to 2012, during which global food prices, although there is a downward trend, are still higher than the values in other years. The third period is the COVID-19 pandemic. The global food price index initially exhibited a modest decline, followed by a resurgence at the end of January 2022. The fourth period is after the start of the Russia–Ukraine conflict, and the global food price index depicted a minor peak, indicating further fluctuations in market dynamics [42].

**Figure 6:**
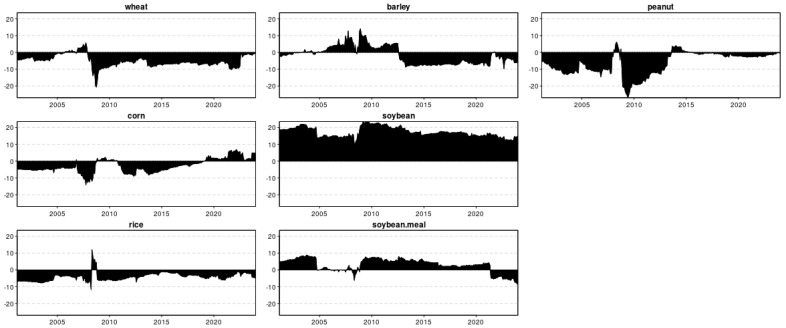
Dynamic net total directional connectedness (NET).

Figure 6 depicts the dynamic net total directional connectedness, signifying the influence emanating from this particular time series to others, subtracting the influence received from other time series, which means the influence the specific grain price has on the analyzed network of selected grain markets. An analysis of the total connectedness index indicates that soybean consistently acts as the most persistent net transmitter of spillover shocks in terms of overall net connection. Research shows that during various turmoil periods—including the 2008–2009 global financial crisis, the 2011–2012 food crisis, the 2020 COVID-19 pandemic, and the Russia–Ukraine war since 2022—soybean price net spillover indices often dominate those of other grains, particularly with positive values. This suggests that soybeans play a leading role in information transmission among these grain markets. Conversely, wheat, rice, corn, and peanut prices consistently demonstrate negative net spillover indices, signifying their status as net receivers of information throughout most of the observed periods. Fluctuation peaks observed during 2007–2008 are likely attributable to sharp increases in oil prices, which impact agricultural commodity prices, particularly grains, given their use as inputs in renewable energy production [52]. Given the inherent challenges of rapidly expanding the supply of agricultural commodities to meet rising energy demands, the need to balance primary agricultural uses with this secondary demand is likely to result in increased price levels for agricultural commodities [53,54]. The diminished prominence of peanuts during 2008–2012 can be attributed to the global food crisis. Additionally, grains play a pivotal role in the production of biofuels, such as fuel ethanol, which are integral components of clean energy initiatives [55]. In addition, the spillover transmission of wheat exhibits a declining trend, while barley demonstrates rapid increases during 2021–2023, which could be influenced by the COVID-19 pandemic and the Russia–Ukraine conflict [52]. The COVID-19 outbreak greatly disrupted the grain industry and the wider economy, driven by reduced demand, business closures, supply chain interruptions, energy crises, panic buying, containment measures, social distancing and trade disruptions [12]. Furthermore, the Russia–Ukraine conflict affects global wheat markets, as the two countries collectively account for approximately 25% of global wheat exports [56].

**Figure 7:**
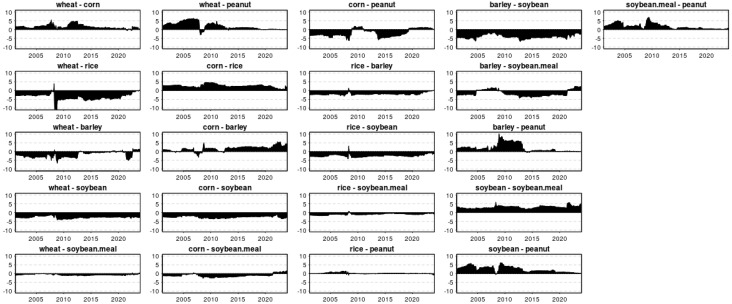
Dynamic net pairwise directional connectedness (PAIR).

Illustrated in Figure 7 are the dynamic patterns of pairwise directional connectedness about grain prices, with positive numerical values denoting a net transmitting role and negative values indicating a net receiving role to examine the bidirectional relationships between different grain pairs. It is intriguing to observe that wheat predominantly dominates corn and peanut in most instances, even during the initial stages of the Russo–Ukrainian War in 2022 and the global financial crisis of 2008, when fluctuations occurred in the relationship between wheat and both corn and peanut. Conversely, wheat itself is typically dominated by rice, barley and soybean, albeit briefly dominated by rice during the 2008 financial crisis. Notably, the 2008 financial crisis also led to a distinct relationship between corn and barley, with corn usually being the dominant force. As depicted in Figure 7, rice typically acts as the primary conduit for shock transmission among crops, being predominantly influenced by barley, soybean, and soybean meal. Although barley generally holds a dominant position in its relationship with peanut, it is typically dominated by soybean and soybean meal. As illustrated in Figure 7, soybean often serves as the primary conduit for shock transmission, exhibiting a strong dominance and proactivity in influencing prices. It is also noteworthy to examine the pairwise bilateral relationships between wheat and soybean meal, rice and peanut, and rice and soybean meal. These relationships are interesting due to the mutual influence between the two crops appears to be symmetrical, as indicated by the near-horizontal alignment in the graph, suggesting that neither party is driving the other.

**Figure 8:**
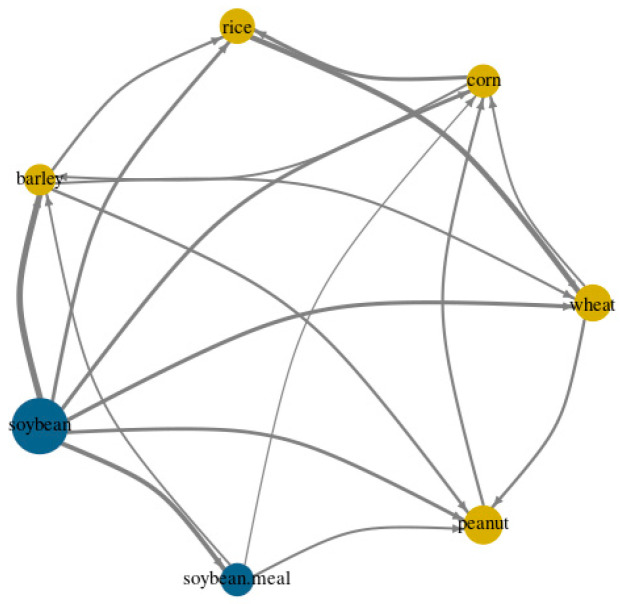
Network diagram for price volatility spillovers.

Figure 8 presents a network diagram illustrating the interconnectedness among grains through pairwise directional returns. In the diagram, arrows represent the flow of spillover, with arrowheads indicating recipient nodes and bases showing transmitter nodes. Recipient nodes are marked in yellow, while transmitter nodes are in blue, highlighting spillover dynamics. Node size reflects the strength of pairwise price linkages. The analysis shows soybean and soybean meal as key transmitters of spillover, with rice, corn, peanut, barley, and wheat as primary recipients. Notably, soybean is a major transmitter to all other grains. In summary, during market fluctuations, the corn price index is particularly complex, influenced by soybean, soybean meal, peanut, and wheat, while soybean significantly impacts the other six grains, especially barley.

## 5. Discussion and Conclusions

In this study, the TVP-VAR-Connectedness approach has been applied to examine the dynamics of price spillover among diverse grain commodities within the international arena. This analytical approach has been instrumental in discerning the roles of net recipients and net transmitters of spillover shocks within the intricate network of different grains. The main conclusions are given as follows.

(1) The employment of a dynamic TVP-VAR-Connectedness methodology has facilitated the identification of both net recipients and transmitters of spillover shocks within the intricate network of grain markets. Notably, soybean prices have been found to exhibit a pronounced spillover effect, suggesting their role as significant net transmitters of price volatility. Conversely, corn and peanut have demonstrated heightened susceptibility to external spillovers, indicating its propensity as a net recipient during periods of market stress.
(2) This study finds that the spillover indices for diverse grain prices peaked in 2022, particularly amidst the Russia–Ukraine conflict and the COVID-19 pandemic. This surge underscores the robust transmission of price shocks across grain markets, with corn and soybeans emerging as the principal conduits for such shocks during these tumultuous times.
(3) A remarkable feature of our research is the emphasis on the interconnected nature of the global grain markets. The potential repercussions of price shocks on various grains are highlighted, underscoring the imperative for the development and implementation of robust risk management strategies to mitigate the adverse effects of market volatility.
(4) We shed light on the escalating challenges to global food security, significantly exacerbated by the confluence of the global food crisis, the COVID-19 pandemic, and the Russia–Ukraine conflict. These factors have collectively contributed to an environment of heightened uncertainty and risk in the global grain markets.
(5) Based on the analysis of dynamic connectedness indices, it has been discerned that the price dynamics of corn exhibit the highest degree of complexity, being significantly influenced by the price indices of soybean, soybean meal, peanut, and wheat. Furthermore, the price index of soybean exerts a notable influence on the remaining six grains within this study, with a particularly pronounced effect on barley.

Our unique perspective provides a more accurate representation of the complex dynamics inherent in grain markets, offering a quantitative method to assess spillover intensity and direction. By studying grain price volatility across varieties, our research contributes to a comprehensive understanding of the implications of international grain price fluctuations on investment decisions, aiding in risk management and optimizing spatial transmission. However, conducting further research regarding the identification of key factors impacting interconnectedness to provide valuable insights for policymakers to ensure stability in the grain market is necessary in the future. The central inquiry of this study is directed towards examining the phenomenon of price volatility spillover and the intricate time-varying characteristics of direction, intensity, and connected network of volatility spillover. The future trajectory of these price fluctuations over the next few years, while not constituting the primary scope of this investigation, nonetheless presents a compelling avenue for future research that warrants an in-depth exploration. So, it would be an insightful study to predict the price dynamics and volatility spillover and visually indicate how these prices tend to vary over the next few years in our future research. Asymmetry in the price transmission and volatility spillovers is common for international grain markets, and it may be possible to employ asymmetric TVP-VAR for analyzing the asymmetric characteristics to figure out price leadership relation.

## Figures and Tables

**Table 1:** ADF test results of time series of prices of seven selected grains.

		ADF Statistic	*p*-Value	ADF Statistic	*p*-Value	Critical Values		
						1%	5%	10%
		Level		First Difference				
wheat	Interception	−2.653	0.084	−13.550	0.000	−3.454	−2.872	−2.572
	both	−2.802	0.198	−13.533	0.000	−3.992	−3.426	−3.136
	none	−0.517	0.492	−13.566	0.000	−2.573	−1.942	−1.616
corn	Interception	−2.201	0.207	−12.862	0.000	−3.454	−2.872	−2.572
	both	−2.401	0.378	−12.850	0.000	−3.992	−3.426	−3.136
	none	−0.388	0.544	−12.875	0.000	−2.573	−1.942	−1.616
rice	Interception	−2.399	0.143	−11.698	0.000	−3.454	−2.872	−2.572
	both	−2.644	0.261	−11.676	0.000	−3.992	−3.426	−3.136
	none	−0.111	0.645	−11.689	0.000	−2.573	−1.942	−1.616
barley	Interception	−2.564	0.102	−11.041	0.000	−3.454	−2.872	−2.572
	both	−2.541	0.308	−11.030	0.000	−3.992	−3.426	−3.136
	none	−0.571	0.469	−11.056	0.000	−2.573	−1.942	−1.616
soybean	Interception	−2.490	0.119	−11.621	0.000	−3.454	−2.872	−2.572
	both	−2.981	0.139	−11.606	0.000	−3.992	−3.426	−3.136
	none	−0.230	0.603	−11.621	0.000	−2.573	−1.942	−1.616
peanut	Interception	−1.419	0.573	−7.594	0.000	−3.454	−2.872	−2.572
	both	−3.325	0.064	−7.578	0.000	−3.992	−3.426	−3.136
	none	0.433	0.807	−7.528	0.000	−2.573	−1.942	−1.616
soybean meal	Interception	−2.721	0.072	−12.520	0.000	−3.454	−2.872	−2.572
	both	−3.732	0.022	−12.498	0.000	−3.992	−3.426	−3.136
	none	−0.303	0.576	−12.527	0.000	−2.573	−1.942	−1.616

**Table 2:** Summary statistics of time series of key grains.

	Wheat	Corn	Rice	Barley	Soybean	Soybean Meal	Peanut
Mean	0.667	0.669	0.493	0.673	0.699	0.736	0.614
Variance	0.554	0.551	0.757	0.545	0.510	0.456	0.622
Skewness	2.265 ***	2.138 ***	6.702 ***	2.291 ***	1.782 ***	1.464 ***	2.985 ***
	0.000	0.000	0.000	0.000	0.000	0.000	0.000
Ex. Kurtosis	6.563 ***	6.127 ***	61.662 ***	6.702 ***	3.895 ***	2.432 ***	12.486 ***
	0.000	0.000	0.000	0.000	0.000	0.000	0.000
JB	728.598 **	639.731 **	45,625.763 *	755.301 **	319.392 **	165.961 **	2194.767 **
	0.000	0.000	0.000	0.000	0.000	0.000	0.000
ERS	−3.979 ***	−3.607 ***	−4.339 ***	−5.068 ***	−4.743 ***	−5.287 ***	−3.830 ***
	0.000	0.000	0.000	0.000	0.000	0.000	0.000
Q (10)	63.112 ***	50.297 ***	126.239 ***	87.349 ***	40.191 ***	42.077 ***	116.717 ***
	0.000	0.000	0.000	0.000	0.000	0.000	0.000
Q2 (10)	38.774 ***	12.380 **	41.925 ***	43.681 ***	31.242 ***	19.395 ***	67.430 ***
	0.000	−0.022	0.000	0.000	0.000	0.000	0.000

* *p*-value < 0.1, ** *p*-value < 0.05, *** *p*-value < 0.001.

**Table 3:** Results of average connectedness.

	Wheat	Corn	Rice	Barley	Soybean	Soybean Meal	Peanut	FROM
wheat	57.470	8.490	12.350	8.320	8.450	4.350	0.580	42.530
corn	10.080	49.220	5.220	8.360	13.820	10.080	3.220	50.780
rice	8.740	7.850	72.550	3.290	4.430	2.830	0.310	27.450
barley	6.560	10.070	1.350	57.340	14.290	9.530	0.860	42.660
soybean	5.750	11.240	2.030	10.190	41.410	28.710	0.660	58.590
soybean meal	3.690	9.010	1.860	8.000	31.810	44.870	0.760	55.130
peanut	2.570	1.300	0.470	2.890	3.070	2.530	87.190	12.810
TO	37.380	47.960	23.290	41.040	75.870	58.030	6.380	289.960
Inc. Own	94.850	97.180	95.840	98.380	117.290	102.890	93.570	cTCI/TCI
NET	−5.150	−2.820	−4.160	−1.620	17.290	2.890	−6.430	48.33/41.42
NPT	2.000	2.000	2.000	3.000	6.000	5.000	1.000	

## Data Availability

The original contributions presented in the study are included in the article, further inquiries can be directed to the corresponding authors.
